# Kale Attenuates Inflammation and Modulates Gut Microbial Composition and Function in C57BL/6J Mice with Diet-Induced Obesity

**DOI:** 10.3390/microorganisms9020238

**Published:** 2021-01-24

**Authors:** Md Shahinozzaman, Samnhita Raychaudhuri, Si Fan, Diana N. Obanda

**Affiliations:** Department of Nutrition and Food Sciences, University of Maryland, College Park, MD 20742, USA; mshahin@umd.edu (M.S.); samnhita@umd.edu (S.R.); sfan9@umd.edu (S.F.)

**Keywords:** kale vegetable, inflammation, *Coriobacteriaceae*, glycan degradation, thiamine metabolism

## Abstract

Kale (*Brassica oleracea* var. *acephala*) is a vegetable common in most cultures but is less studied as a functional food compared to other cruciferous vegetables, such as broccoli. We investigated the effect of supplementing a high-fat diet (HFD) with kale (HFKV) in C57BL/6J mice. We particularly explored its role in metabolic parameters, gut bacterial composition and diversity using 16S rRNA sequencing, systematically compared changes under each phylum and predicted the functional potential of the altered bacterial community using PICRUSt2. Like other cruciferous vegetables, kale attenuated HFD-induced inflammation. In addition, kale modulated HFD-induced changes in cecal microbiota composition. The HFD lowered bacterial diversity, increased the Firmicutes: Bacteroidetes (F/B) ratio and altered composition. Specifically, it lowered Actinobacteria and Bacteroidetes (Bacteroidia, Rikenellaceae and Prevotellaceae) but increased Firmicutes (mainly class Bacilli). Kale supplementation lowered the F/B ratio, increased both alpha and beta diversity and reduced class Bacilli and Erysipelotrichi but had no effect on Clostridia. Within Actinobacteria, HFKV particularly increased Coriobacteriales/*Coriobacteriaceae* about four-fold compared to the HFD (*p* < 0.05). Among Bacteroidia, HFKV increased the species *Bacteroides thetaiotaomicron* by over two-fold (*p* = 0.05) compared to the HFD. This species produces plant polysaccharide digesting enzymes. Compared to the HFD, kale supplementation enhanced several bacterial metabolic functions, including glycan degradation, thiamine metabolism and xenobiotic metabolism. Our findings provide evidence that kale is a functional food that modulates the microbiota and changes in inflammation phenotype.

## 1. Introduction

Diet has increasingly become a key strategy for preventing disease and improving health conditions. In this context, cruciferous vegetables (genus *Brassica*) have been investigated for several years. Studies in animal models, human trials and epidemiological studies show evidence that diets rich in cruciferous vegetables are associated with better health outcomes and a lower risk of non-communicable diseases, such as cancer and metabolic diseases [1,2]. Broccoli, cabbage, cauliflower and brussels sprouts are the most popular brassica crops. For several years, broccoli has been the most widely studied and widely reviewed cruciferous vegetable. Although less studied, kale (*Brassica oleracea* var. *acephala*), traditionally a less recognized member of the genus Brassica, has gained popularity in the United States in recent years. It has become an important crop in the southern US as a result of its suitability to year-round growing conditions [1]. Similar to other cruciferous vegetables, phytochemicals in kale include sulfur-containing indolic glucosinolates and aliphatic glucosinolates, polyphenols like the flavonoid glycosides of quercetin, kaempferol and isorhamnetin and carotenoid groups [3,4,5,6].

Kale ranks high on the list of the most healthy foods or super foods. In a study that developed a classification scheme defining powerhouse fruits and vegetables as foods providing 10% or more of the daily value per 100 kcal of 17 qualifying nutrients, kale was ranked 15 out of the 41 foods that satisfied the powerhouse criterion [7]. No studies on kale as a functional food appear to have been carried out in the last few decades. It is unclear how kale compares to other cruciferous vegetables as a functional food, particularly how it impacts the gut microbiota.

Both basic and clinical research continue to show the connection between diet and the composition and health of the gut microbiota. The gut microbiota influences host phenotype through contact with intestinal cells. Bacterial metabolites from dietary ingredients may indirectly impact host cellular mechanisms. Diet alters both gut microbiota population and diversity. Diet- or obesity-induced dysregulation in microbiota health can be attenuated by manipulating the microbiota to target the proliferation of bacterial species that influence positive metabolic functions. Prebiotic foods induce the proliferation of beneficial bacteria and gut bacteria may metabolize the prebiotic food components to produce molecules that impact host mechanisms. Prebiotic vegetables which promote microbiota health and diversity include chicory, Jerusalem artichokes and garlic, which all have a high contents of inulin-type fructan, oligofructose and inulin [8,9]. Broccoli consumption has been shown to lower phylum Firmicutes, increase phylum Bacteriodetes and improve hydrolysis of glucosinolates [10]. The effects of kale on gut microbiota have not been studied before.

The high proportion of soluble and insoluble fiber and phytochemicals in kale may affect gut bacteria species composition and diversity, serve as a substrate for gut bacteria and result in the proliferation of beneficial bacterial taxa or metabolites that influence host cellular mechanisms. Herein, we induced dysbiosis, inflammation and obesity in C57BL/6J mice using a high-fat diet (HFD) and investigated how kale prevents or attenuates these conditions and, particularly, how it impacts bacterial composition and diversity with ultimate benefits to the host. We systematically analyzed changes within each phylum. We report that supplementing the HFD with kale increases bacterial diversity and impacts the community structure of the microbiota and its metabolic function, with the most abundant changes attributed to the abundance of bacteria within the family *Coriobacteriaceae* and the species *B. thetaiotaomicron* and their metabolic functions.

## 2. Materials and Methods

### 2.1. Study Animals and Study Diets

All procedures were in accordance with a protocol approved by the Institutional Animal Care and Use Committee (IACUC) of the University of Maryland (Approval Ref: R-JUL-19-36). Twenty-seven (27) male C57BL/6J mice at 7 weeks old (Jackson Laboratories, Inc., Bar Harbor, ME, USA) were singly housed and maintained in light/dark cycles of 12:12 h at 22 ± 1 °C. Curly green kale sourced from the farmers’ market at the University of Maryland was oven dried (50 °C) and powdered using a coffee grinder. Nutrition analysis by Association of Analytical Chemists (AOAC) methods determined: moisture, ash, total lipid, protein, carbohydrate, soluble fiber, insoluble fiber and fatty acid profile. A list of methods used is shown in Table 1.

All diets were formulated to be isocaloric (3961 kcal/kg) with equal sucrose and protein (Table 2). The low-fat diet (LFD) control contained 10% fat, the HFD and high-fat diet with kale (HFKV) contained 45% fat. The 9% kale was equivalent to 0.23 g per day for a 30 g mouse. Assuming a human consumes 4.1 lbs (1.81 kg) food per day, that translates into 0.16 kg of kale per day.

### 2.2. Randomization and Sample Size Determination

Based on body weight and the homeostatic model assessment of insulin resistance (HOMA-IR), mice were randomized onto the 3 diet groups for 12 weeks with food and water provided ad libitum. A sample size of *n* = 9 was used based on a power analysis in our previous animal study on obesity-induced insulin resistance, which showed 9 to be a number sufficient to generate statistically significant results and attain a statistical power of 0.90 for a 2-tailed test.

### 2.3. Determination of Body Weight and Insulin Sensitivity

Body weight and food intake were recorded twice a week. Fasting (6 h) plasma glucose and insulin were determined at baseline and at 6 and 12 weeks. Blood glucose was measured by a portable glucometer and strips. Insulin levels were determined by a mouse ELISA kit (Crystal Chem, Downers Grove, IL, USA). Insulin resistance (HOMA-IR index) was calculated as shown before in Roza et al. [11].

### 2.4. Euthanasia, Determination of Fat Accumulation and Tissue Collection

After 12 weeks, animals were anesthetized by isoflurane inhalation. Terminal blood was collected by heart puncture followed by euthanasia by cervical dislocation. The weights of the epididymal, perirenal and retroperitoneal fat pads were summed as total abdominal fat. All collected tissues, cecum contents and colon fecal samples were snap frozen in liquid nitrogen and later stored at −80 °C.

### 2.5. Determination of Fermentation Variables

At euthanasia, the weight of the full cecum and empty cecum was determined. The pH of colon fecal samples from the central section (next to the cecum) was determined; briefly, 1 g of colon fecal contents was mixed with 500 μL deionized water, and centrifuged at 13,000× *g* for 2 min. The pH of the supernatant was determined using an Orion Dual Star pH meter (Thermofisher Scientific, Rockville, MD, USA). Cecum weight is a marker of fermentation because the weight and volume of the cecum increases with fermentation as the total amount of bacteria in the cecum increases and products of fermentation, like short chain fatty acids, increase in quantity. The pH is also a marker of fermentation because higher levels of fermentation result in more short chain fatty acids, which lower pH [12].

### 2.6. RNA Extraction and cDNA Synthesis

RNA from 100 mg adipose (epididymal fat pad) was separately extracted and purified using the RNAeasy mini kit (Qiagen, German Town, MD, USA) according to the manufacturer’s specifications. Prior to extraction, the samples were disrupted in TRIzol with bead beating using a FastPrep-24 (MP Biomedical, Solon, OH, USA). Both RNA quantity and quality were determined using a Qubit 4 fluorometer (Thermofisher Scientific, Rockville, MD, USA). Only RNA with an integrity number greater than 7.8 was used in cDNA synthesis for qPCR. Similarly, only RNA samples with a concentration over 2.3 ng/µL and an RNA integrity number greater than 7.8 were used for cDNA synthesis using the RT^2^ first strand kit (Qiagen, German Town, MD, USA) with 1000 ng as starting RNA per sample.

### 2.7. Analysis of Target Genes in Adipose Tissue by qPCR

Primer sets (IDT Technologies, Coralville, IA, USA) were used for analyses of inflammatory cytokine genes. RT-PCR cycling conditions on a CFX 96 (Bio-rad, Hercules, CA, USA) were 2 min at 50 °C and 2 min at 95 °C, followed by 40 cycles of two-step PCR denaturation at 95 °C for 15 s and annealing extension at 60 °C for 1 min. Duplicate assay samples contained 10 ng cDNA and 6 μmol/L primers in 2× PowerUp SYBR Green Master Mix (Thermofisher Scientific) in a final volume of 20 μL. Means of duplicates were taken, and the relative amount of target mRNA in each sample was normalized to β-actin levels as an endogenous control gene. Data were analyzed according to the 2^−ΔΔ*C*t^ method, and fold difference was calculated between groups.

### 2.8. DNA Extraction

Genomic DNA was extracted from 100 mg of cecal contents using the PowerFecal Pro DNA kit (Qiagen, Germantown, MD, USA) according to the manufacturer’s instructions. Prior to DNA extraction, samples were disrupted in 2 mL tubes with bead beating using a FastPrep-24 (MP Biomedical, Solon, OH, USA). DNA samples were stored at −80 °C to be used in sequencing. DNA quantity was determined using a Qubit 4.0 Fluorometer (ThermoFisher Scientific, Rockville, MD, USA).

### 2.9. Amplification, Library Preparation, Template Preparation and Sequencing

Seven hypervariable regions of the 16S rRNA gene (V2, V3, V4, V6, V7, V8 and V9) were amplified by PCR using the Ion 16S™ Metagenomics kit (Thermofisher Scientific, Rockville, MD, USA). Libraries were prepared and bar-coded (ligation) using end repair buffer and enzymes contained in the Ion Plus Fragment Library kit and the Ion Xpress™ Barcode Adapters 1–16 kit and 17–32 kit (Thermofisher Scientific, Rockville, MD, USA). Libraries were diluted to yield 10 pM input for template preparation and enrichment using an Ion OneTouch™ 2 System and the Ion PGM™ Hi-Q™ OT2 Kit. Template-positive Ion PGM™ Hi-Q™ Ion Sphere™ Particles (ISPs) with 400 base pair average insert libraries were sequenced on an Ion 530™ Chip using the Ion GeneStudio™ S5 system (ThermoFisher Scientific, Grand Island, NY, USA).

### 2.10. Microbial Diversity Analysis and Bioinformatics

The Ion Torrent Suite software package was used to remove low-quality and polyclonal sequences, and the remaining reads were analyzed using QIIME2 [13]. Sequences were clustered into operational taxonomic units (OTUs) using closed-reference OTU picking against the Greengenes v13.5 and MicroSEQ Library v2013.1 reference database with a 97% similarity threshold. Each OTU could be presented as counts, percent total reads and percent of mapped reads. For this paper, all subsequent comparisons within groups were performed using percent of mapped reads. The OTU table, which indicates the number of reads per sample, was used for the subsequent statistical analysis. After quality filtering, alpha diversity and beta diversity were calculated at an even sampling depth of 424,226 sequences per sample. For alpha diversity, the observed species, Shannon and Simpson indices were calculated for each data set. Alpha rarefaction plots were produced by plotting the number of sequences against the respective index. For beta diversity, principal component analysis (PCA) was performed with mean centered data using R software (version 4.0.2, R core Team, Vienna, Austria). Permutational multivariate analysis of variance (PERMANOVA) was performed by “QIIME diversity beta-group-significance” command using Bray-Curtis distance between samples with 999 permutations.

### 2.11. Analysis of Enrichment of Bacteria in Each Study Group

We used linear discriminant analysis effect size (LEfSe) to determine the OTUs and clades most likely to explain differences between the LFD, HFD and HFKV groups with additional tests for biological consistency and effect relevance, as shown by Segata et al. [14]. The LEfSe analysis was done with the abundance information file of all bacteria obtained at all OTU levels. We obtained *p*-values for the factorial Kruskal–Wallis test among treatment groups and obtained *p*-values for the pairwise Wilcoxon rankings with an linear discriminant analysis (LDA) score >4 for any significant taxa between subclasses. Results were analyzed for false discovery rate by Benjamini–Hochberg tests where only *p*-values < 0.05 and *q*-values < 0.25 were considered significant for an association between metadata and taxonomy.

### 2.12. PICRUSt2

Phylogenetic Investigation of Communities by Reconstruction of Unobserved States (PICRUSt2) analysis was performed from sequencing data, as shown before [15] to predict the metabolic pathways. Demultiplexed and trimmed Fastq sequence files for each sample were curated from Ion Reporter and processed using QIIME2. Quality filtered demultiplexed sequences were denoised by removing chimeric sequences and correcting amplicon errors using the DADA2 plugin. Representative sequences and the feature table generated by DADA2 were used as input files for the PICRUSt2 analysis pipeline. Metabolic pathways were assigned based on the Kyoto Encyclopedia of Genes and Genomes (KEGG) Ortholog (KO) database. Read abundance data for all predicted pathways were converted to relative abundance (%). Data were then processed and analyzed by R software (version 4.0.2) to prepare PCA plots and by the Galaxy server (https://huttenhower.sph.harvard.edu/galaxy/) for LEfSe analysis using LDA score 2.5 as a threshold level.

## 3. Results

### 3.1. Body Weight, Fat Accumulation, Insulin Sensitivity and Inflammation

Weekly food intake, final body weight, abdominal fat weight, insulin resistance (HOMA-IR) and inflammation markers are shown in Table 3a. Calorie intake was not different among the three groups but the HFD induced significantly higher body weight, insulin resistance and inflammation compared to the LFD. Body weight, total abdominal fat and HOMA-IR trended lower in the HFKV group compared to the HFD group but they were not statistically different (Table 3a). However, HFKV significantly reduced the expression of inflammatory marker MCP-1 in serum (*p* < 0.05) and increased IL-10 (*p* < 0.05; Table 3a). In adipose tissue (epidydimal fat pad), HFKV attenuated the HFD-induced mRNA expression of several markers of inflammation, including TNFα, MCP-1, CD11c, F4/80 and IL-6 (*p* < 0.05; Table 3b). The primers used to determine the expression of these markers are shown in Table 3b. Cytokine IL-8 was not detected in these samples, while IL-1β and NF-κB expression between the HFD and HFKV groups was not different (Table 3b).

### 3.2. Markers of Fermentation

The weights of the full cecum and weight of the empty cecum were not significantly different between LFD, HFD and HFKV diets. The pH of the colon fecal samples was also not different between all the diets (Table 4). Colon fecal samples from the proximal part of the colon (next to the cecum) were used for pH determination because most of the cecum contents were used up in the extraction of bacterial DNA.

### 3.3. Diversity Metrics

**Figure 1:**
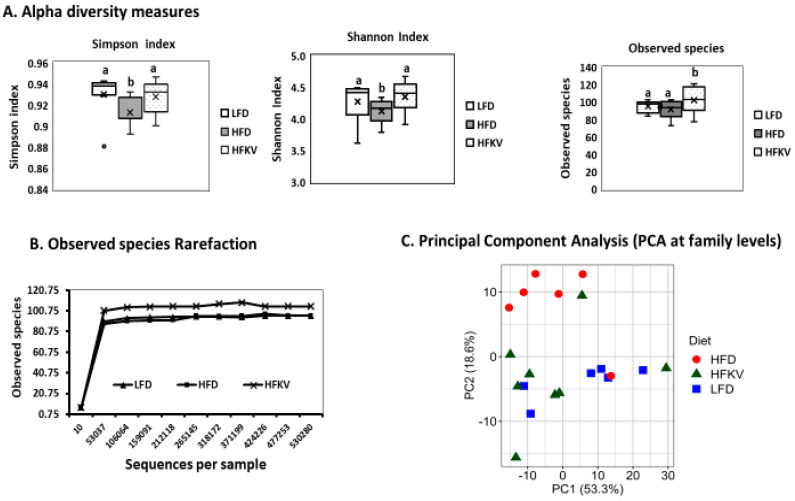
Alpha and beta diversity measures. (**A**) Alpha diversity plots. Boxes represent the interquartile range between the first and third quartiles. The horizontal line inside the box defines the median and the “x” represents the mean. Whiskers represent the lowest and highest values within 1.5 times the interquartile range between the first and third quartiles, respectively. The dots outside the box represent outliers. All diversity indices were higher in the HFKV group compared to both LFD and HFD (*p* < 0.05). The letters ‘a’ and ‘b’ denote statistically significant values at *p* < 0.05; (**B**) The rarefaction curve reached a summit after 53,037 sequences for all treatment groups, as shown by the alpha diversity (Shannon analysis); (**C**) Principal component analysis (PCA) score plot of beta diversity. The different colors represent different diet groups, and the closer data points represent similarity in microbial composition (Pseudo F = 4.11; *p* = 0.001).

The alpha diversity of the microbiota of HFKV fed mice was significantly higher than those of the LFD and HFD, as assessed by measures of species richness: observed species (count of unique OTUs in each sample). Shannon and Simpson indices reflect the evenness and richness of the various OTUs (*p* < 0.05) (Figure 1A). The greater the indices, the higher the diversity. The rarefaction curves reached a summit in each sample, indicating that the sequencing depth was sufficient to detect all the genera within each sample and suitable to capture the microbial diversity (Figure 1B). PCA showed that the first two components, PC1 and PC2, explain 53.3% and 18.6% of the total variance, respectively. As shown in the PCA score plots (Figure 1C), LFD and HFKV samples are mostly distributed in a particular PC direction, while HFD samples are scattered in a different dimension. Overall, PCA revealed that although the LFD, HFD and HFKV groups show some degree of similarity in their gut microbiota structure with overlapping clusters, the HFKV diet increased microbial diversity by clustering in different directions. A total 71.9% variances are covered by principal components 1 and 2. The LFD and HFKV groups shared more similarity (Figure 1C). Overall, PCA reveals that the HFKV diet increased gut microbial diversity compared to the LFD and HFD. PERMANOVA revealed significant differences between the diets (PseudoF = 4.11; *p* = 0.001).

### 3.4. Comparative Analysis of the Gut Microbial Composition

**Figure 2:**
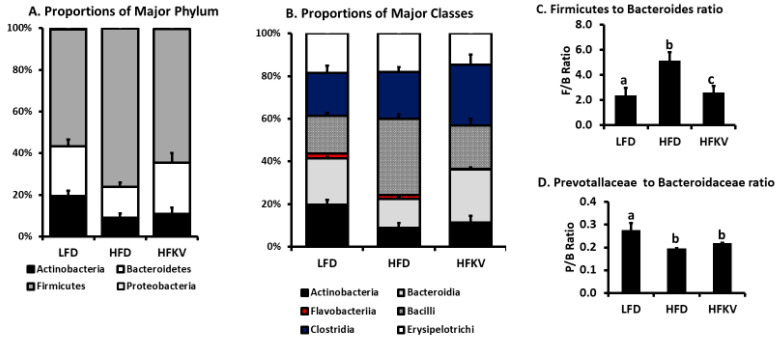
Histograms of relative abundance. (**A**) Relative abundance of the top 4 phyla. The *Y*-axis represents relative abundance presented as a percentage; (**B**) Relative abundance of the top 6 classes; (**C**) Ratio of Firmicutes to Bacteroides was significantly higher in HFD compared to LFD (*p* < 0.05) and HFKV lowered the ratio to levels of the LFD (*p* < 0.05); (**D**) Ratio of Prevotallaceae to Bacteroidaceae was lower in the HFD compared to LFD (*p* < 0.05) and HFKV had no significant effect (*p* = ns). The letters ‘a’ ‘b’ ‘c’ denote statistically significant values at *p* < 0.05.

As expected at the phylum level, Firmicutes was the most abundant, followed by Bacteroidetes and Actinobacteria. The HFD reduced Actinobacteria and Bacteroidetes and increased Firmicutes (*p* < 0.01) (Figure 2A). The HFKV diet had no effect on Actinobacteria but increased Bacteroidetes while decreasing Firmicutes (*p* < 0.01). The most abundant classes were Actinobacteria, Bacteroidia, Bacilli, Clostridia and Erysipelotrichi, while Flavobacteria was present in much lower proportions (Figure 2B). Flavobacteria was significantly reduced in HFKV compared to both the LFD and HFD (*p* < 0.005). Compared to the LFD, the HFD reduced classes Actinobacteria and Bacteroidia but increased Bacilli (*p* < 0.05). HFKV reduced Bacilli and increased Bacteroidia (*p* < 0.05) but had no effect on Actinobacteria (*p* = ns) (Figure 2B). Overall, the ratio of Firmicutes to Bacteroidetes was increased by the HFD compared to the LFD. HFKV reduced the ratio of Firmicutes to Bacteroidetes to levels similar to that of the LFD (Figure 2C).

### 3.5. Comparative Analysis of Phylum Actinobacteria

The HFD lowered phylum Actinobacteria by 56.8% and class Actinobacteria by about 58% compared to the LFD (*p* < 0.05, *n* = 9). The HFKV diet had no effect; did not prevent or reverse these effects (Table 5). Among class Actinobacteria, the most prominent order, comprising 90%, was Bifidobacteriales along with genus Bifidobacterium, while order Coriobacteriales constituted about 1–4%. Furthermore, diversity among Bifidobacterium was low, with the bulk of it being constituted by the species *B. pseudolongum*. The HFD lowered this species and HFKV did not reverse or attenuate the reduction. *B. choerinum* was also detected at lower levels with no effect of diet observed on its abundance. The HFD lowered order Coriobacteriales and family *Coriobacteriaceae* compared to the LFD. The HFKV diet increased Coriobacteriales/*Coriobacteriaceae* about four-fold (over 400% increase) compared to the HFD (Table 5). The prominent genera among *Coriobacteriaceae* were *Atopobium* and *Enterorhabdus* and the HFD lowered both significantly compared to the LFD (*p* < 0.05). The HFKV increased them significantly compared to both LFD and HFD (*p* < 0.05) (Table 5).

### 3.6. Comparative Analysis of Phylum Bacteroidetes

The HFD lowered phylum Bacteroidetes by 40.7% compared to the LFD (*p* < 0.05) and HFKV increased it by 42% (*p* < 0.05, *n* = 9). The bulk of this phylum was class Bacteroidia, with class Flavobacteria constituting a much smaller amount (1–3%) (Table 5). The HFD significantly lowered class Bacteroidia by 40.2% and order Bacteroidales by 34.7% compared to the LFD (Table 5). The HFKV diet significantly increased these groups. Particularly, it increased Bacteroidia by 79.0% and Bacteroidales by 79.2% compared to the HFD (*p* < 0.001) (Table 5). The HFD also had no effect on the classes Flavobacteria and Sphingobacteria but reduced Cytophagia compared to the LFD. HFKV lowered Flavobacteria but increased Sphingobacteria compared to the HFD (*p* < 0.05). Class Cytophagia and Sphingobacteria constituted 0.3% and 0.05% of the bacteria, respectively (Table 5).

Among order Bacteroidales, the most prominent family was Bacterioidaceae, constituting about 60–80%. The HFD lowered Bacterioidaceae by 30.7%, while HFKV increased it by 79.4% (*p* < 0.001). Among Bacterioidaceae, diversity was low; about 100% of this family constituted genus *Bacteroides* (Table 5). Minor families were Porphyromonadaceae, Prevotellaceae and Rikenellaceae. The HFD significantly lowered these three families compared to LFD levels. The HFKV diet had no effect on Porphyromonadaceae and Rikenellaceae but increased Prevotellaceae compared to the HFD (Table 5). However, the decrease in the Prevotellaceae/Bacteroidaceae ratio induced by the HFD was not attenuated by HFKV (Figure 2D; *p* = ns). Among Rikenellaceae, the HFD had no effect on *Alistipes* proportions compared to LFD levels. The HFKV diet increased *Alistipes* by about 50% compared to the HFD and LFD. Among genus *Bacteroides*, diversity was low and the species *Bacteroides thetaiotaomicron* constituted the bulk of it. The HFD lowered this species by 42.1% (Table 5; *p* < 0.05) while the HFKV diet increased it by about 200% (about two-fold) compared to the HFD proportions (*p* < 0.05).

### 3.7. Comparative Analysis of Phylum Firmicutes

The HFD increased Firmicutes by 28% (*p* < 0.05) compared to LFD. The HFKV significantly lowered this; it reduced Firmicutes by 13% compared to HFD (*p* < 0.05). However, most changes by HFKV in Firmicutes were in the class Bacilli. The HFD increased class Bacilli by 94% (*p* < 0.001) and Clostridia by 3.3% (*p* = ns) compared to the LFD. The HFKV diet decreased Bacilli by 54% compared to the HFD (*p* < 0.05) but further increased Clostridia by 33% compared to the HFD (*p* = ns) (Table 5). A more detailed analysis showed that among class Bacilli, the changes were mainly in order Lactobacillales, with no changes observed in the order Bacillales. Among order Lactobacillales, the HFD increased family Lactobacillaceae and Streptococcaceae (*p* < 0.05) and the HFKV lowered both families (Table 5). Among Streptococcaceae, the most dominant taxa were genus *Lactococcus*, the bulk of which constituted *Lactococcus lactis*. HFKV lowered their abundance to levels similar to those of the LFD group. The most dominant genus among Lactobacillaceae was *Lactobacillus*, particularly the species *Lactobacillus johnsonii*, and HFKV lowered their abundance to levels similar to those of the LFD group (Table 5).

The HFD had no effect on class Clostridia and its family Clostridiaceae and genus *Clostridium* when compared to the LFD. The HFKV diet had no significant effect on these taxa compared to both the LFD and HFD (Table 5). The most prominent species among Clostridia were *C. vincentii* and *C. disporicum* and the HFKV had no effect on their proliferation (Table 5). Among Clostridia, the HFD increased family Lachnospiraceae and HFKV increased it even more compared to the HFD (Table 5). Members of Lachnospiraceae increased by HFKV include Acetatifactor, Rumnococcus, Blautia and Roseburia (Table 5).

The HFD reduced the representation of class Erysipelotrichi compared to the LFD (*p* < 0.05) and HFKV had no significant effects compared to the HFD (*p* = ns) (Table 5). HFKV overall had no significant effect on Erysipelotrichaceae when compared to the LFD (*p* < 0.05). Only one genus (*Turicibacter*) was identified in this family and the HFD and HFKV had no effects on its abundance (Table 5).

### 3.8. Comparative Analysis of Phylum Proteobacteria and Verrucomicrobia

Proteobacteria constituted less than 1% of all bacteria. The three diets had no significant effect on class Betaproteobacteria (*p* = ns) but the HFD significantly increased class Gammaproteobacteria compared to the LFD. HFKV attenuated the HFD-induced increase in Gammaproteobacteria (*p* < 0.05). The bulk of Betaproteobacteria was constituted by order Burkholderiales and, in turn, this order was mainly constituted by family Sutterellaceae. The bulk of Gammaproteobacteria constituted family Enterobacteriaceae (Table 5).

Among Verrucomicrobia, which constituted about 0.05–0.2% of total bacteria, the HFD increased the representation compared to the LFD (*p* < 0.05) and the HFKV diet had no significant effect (*p* = ns). Phylum Verrucomicrobia constituted mainly the species *Akkermansia municiphila* (about 95%) and about 5% was unidentified species among genus *Akkermansia* (Table 5).

### 3.9. Linear Discriminant Analysis Effect Size (LEfSe)

**Figure 3:**
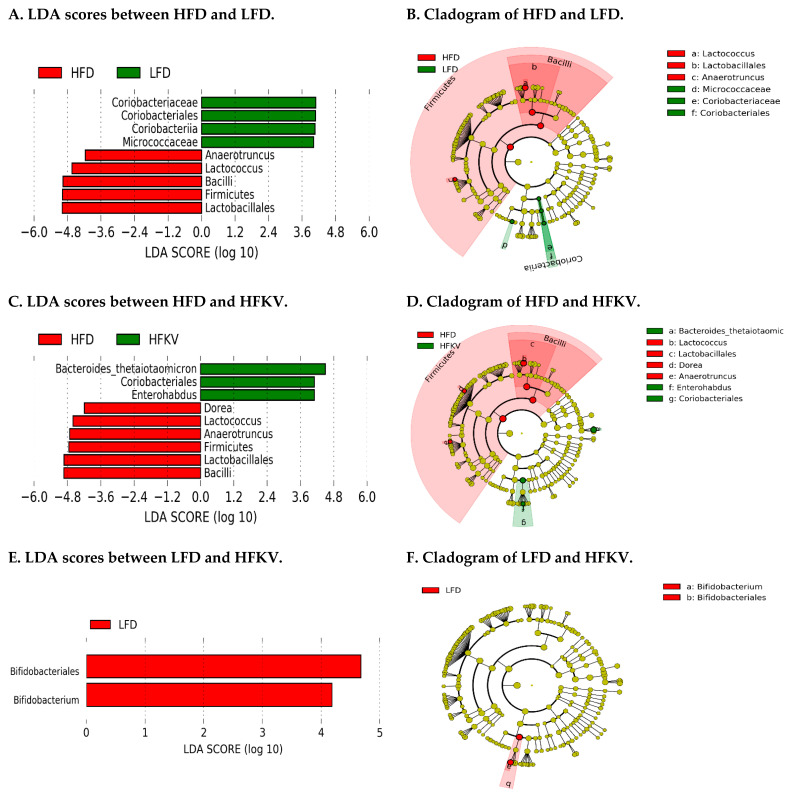
Linear discriminant analysis. (**A**) Linear discriminant analysis (LDA) effect size analysis between LFD and HFD; (**B**) Cladogram of differentially abundant taxonomic clades; LDA score > 4.0 among LFD and HFD; (**C**) Linear discriminant analysis (LDA) effect size analysis between HFD and HFKV; (**D**) Cladogram showing differentially abundant taxonomic clades with an LDA score > 4.0 among HFD and HFKV; (**E**) Linear discriminant analysis (LDA) effect size analysis between LFD and HFKV; (**F**) Cladogram showing differentially abundant taxonomic clades; LDA score > 4.0 among LFD and HFKV.

The data were analyzed using the LEfSe algorithm for high-dimensional biomarker discovery to identify key taxa that account for the differences between the dietary groups. LEfSe estimates the effect size of each significantly different taxon [13]. At a threshold of 4.0 on the logarithmic LDA score, the abundances of nine taxa were different between the LFD and HFD groups and accounted for discriminative features (Figure 3A,B). Nine taxa were different and accounted for discriminative features between the HFD and HFKV (Figure 3C,D) while only two taxa were different between the LFD and HFKV groups and accounted for discriminative features (Figure 3E,F). A *p*-value of < 0.05 was considered significant in Kruskal–Wallis and pairwise Wilcoxon tests.

### 3.10. Predicted Metabolic Functions

**Figure 4:**
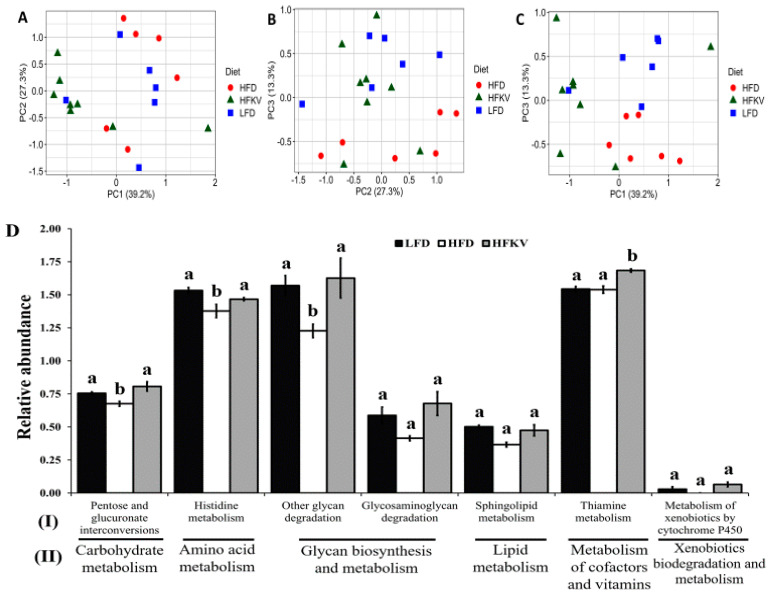
Predicted metabolic pathways by PICRUSt2 using 16s rRNA gene sequences. (**A**–**C**) PCA plots representing the pathways predicted from Kyoto Encyclopedia of Genes and Genomes (KEGG) Ortholog (KO) database. Data points represents the distribution of principal components in two different directions (*x*- and *y*-axis) and the closer the data points, the closer the relationship between the samples. HFKV clustered closer to LFD; (**D**) Microbiota metabolic functions showing significant differences between HFD and HFKV. Significant differences with LFD-induced pathways are indicated by different letters (*p* < 0.05). (I) KEGG pathways at level 1; (II) KEGG pathways at level 2. The letters ‘a’ ‘b’ denote statistically significant values at *p* < 0.05.

PICRUSt2 predicted a total of 152 functional pathways by comparing against KEGG orthologs. Data were transformed to relative abundance and differences between diets are presented in Figure 4. Although PCA plots (Figure 4A–C) show a relatively scattered distribution, HFKV clustered more closely with the LFD group while they clearly show a distinct clustering from the HFD group.

**Figure 5:**
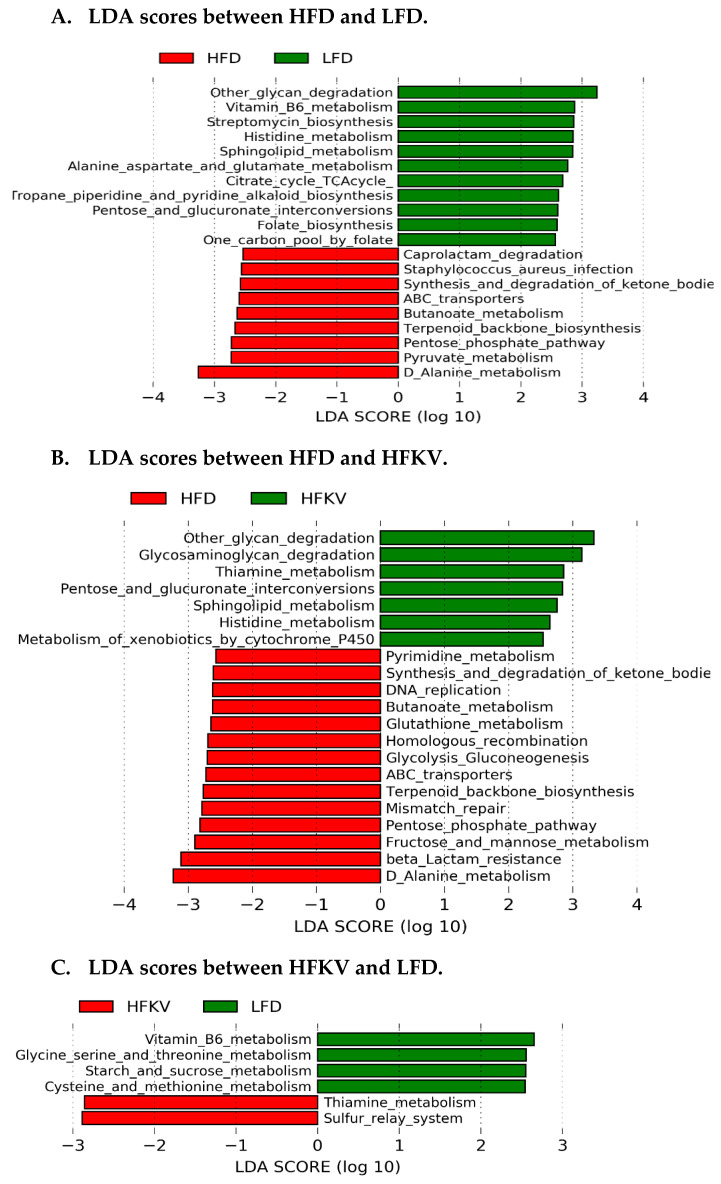
Linear discriminant analysis of predicted microbial metabolic functions. (**A**) Pathways induced by LFD and HFD; (**B**) Pathways induced by HFKV and HFD; (**C**) Pathways induced by LFD and HFKV.

LEfSe analysis at a 2.5 threshold level demonstrates that the LFD and HFD were different in 20 pathways (Figure 5A) and 21 pathways were different and accounted for discriminative features between the HFD and HFKV (Figure 5B), while six pathways were different between the LFD and HFKV (Figure 5C). The HFD significantly reduced three KEGG level 1 metabolic functions; carbohydrate metabolism, glycan biosynthesis and metabolism and amino acid metabolism, compared to the LFD. The HFKV diet reversed the inhibitory effects of the HFD and enriched all these functions (Figure 4D). The HFKV diet increased carbohydrate metabolism, glycan degradation pathways, cofactor and vitamin metabolism and xenobiotics metabolism compared to both the HFD and LFD (*p* < 0.05).

## 4. Discussion

Kale is a cruciferous vegetable that is traditionally part of the diet in many international cultures but has recently become popular in the United States. Despite its gaining popularity, kale remains an understudied vegetable. We show that supplementing an HFD with kale changed microbiota composition, increased both alpha and beta diversity and attenuated the increase in the Firmicutes: Bacteroides (F/B) ratio, a parameter that several studies have shown correlates with lower body weight. However, these factors did not significantly prevent HFD-induced increases in body weight, fat accumulation or insulin resistance. Despite not lowering body weight and fat accumulation, kale supplementation prevented HFD-induced inflammation in adipose tissue. A closer examination revealed that kale induced an increase in bacterial groups of family *Coriobacteriaceae*. The abundance of *Coriobacteriaceae* has been shown to correlate with the abundance of metabolites that underlie improved health outcomes after exercise [16] and treatment for diabetes [17]. Furthermore, kale enhanced microbial functional pathways related to complex carbohydrate and xenobiotic metabolism; pathways that are known to produce metabolites beneficial to host inflammatory mechanisms and the metabolism of plant phytochemicals.

The health benefits of prebiotic vegetables such as chicory and Jerusalem artichokes are attributed to gut bacterial fermentation of their inulin-type fructan and oligofructose component [8,9]. Although the kale sample used in this study contained 6.2% soluble fiber and 37.8% insoluble fiber (Table 1), it did not induce fermentation; no reduced pH of colon fecal samples or increase in weight of the empty and full cecum were observed compared to the LFD and HFD (Table 3). The fiber in kale likely constituted plant polysaccharides that are not fermentable. It is likely that kale phytochemical metabolic products impact the inflammation mechanisms. Like other cruciferous vegetables, the phytochemical composition of kale includes sulfur-containing indolic glucosinolates, aliphatic glucosinolates, polyphenols and carotenoid groups [3,4,5,6] that impact inflammatory mechanisms. All these may individually impact microbiota composition in ways that are yet unknown. The reduction in inflammation by kale mirrors that of broccoli, the most highly studied cruciferous vegetable. Our findings corroborate those of Kaczmarek et al. [10], who showed that broccoli increased bacterial diversity and the abundance of Bacteroidetes and reduced the F/B ratio. The lack of a clear relationship linking the F/B ratio of fecal microbiota to increased body weight or fat accumulation was a surprise. However, when we examined the kale-induced increase in phylum Bacteroidetes more deeply, we found that only the abundance of genus *Bacteroides* was increased. Diversity was low and the increase mainly constituted the species *Bacteroides thetaiotaomicron*. Because the metabolic function of *B. thetaiotaomicron* is to degrade plant polysaccharides, vegetables like kale induce its proliferation among gut bacteria for purposes of producing bacterial plant polysaccharide-digesting enzymes which the host does not produce. This species produces very high levels of digestive enzymes effective in the breakdown and subsequent digestion of plant polysaccharides [18,19]. This may be a contributing factor to why the increased amounts of *Bacteroides* and reduced F/B ratio did not result in reduced body weight or fat accumulation. In the literature, low F/B ratios are associated with reduced blood glucose levels, increased glucose tolerance and low body weight [20]. We show that gut microbiota rich in Bacteroidetes, and *B. thetaiotaomicron* in particular, does not protect mice from developing glucose intolerance and obesity in the HFD feeding condition. The reduction in the F/B ratio was also attributed to fewer Firmicutes in HFKV compared to the HFD. Among Firmicutes, kale supplementation lowered the HFD-induced increase in Bacilli (both Lactobacillaceae and Streptococcaceae) but had no significant effects on Clostridia (Table 5). Species from Clostridia, particularly genus *Clostridium*, have been shown to be increased and correlate with reduced body weight and enhanced insulin resistance through their impact on the intestinal immune system [21,22,23]. The observed lack of kale effects on the abundance of Clostridia may be behind the reason why kale does not impact body weight.

Although Coriobacteriales/*Coriobacteriaceae* constituted only about 1–4% of gut bacteria, the four-fold (over 400%) increase by kale was surprising. *Coriobacteriaceae* includes 30 species belonging to 14 genera. In the gut, *Coriobacteriaceae* carry out important functions, such as conversion of bile salts and steroids and activation of dietary polyphenols, and are key in the production of menaquinone-6 homologs of vitamin K. Several novel species have been described recently and their clinical relevance in host/bacteria interactions is still under investigation [24]. Endurance exercise (long-distance running) and wheel running of animals has been shown to increase the abundance of *Coriobacteriaceae*. This family has been identified as a potential biomarker that links exercise with health improvement and is correlated with the levels of 15 metabolites that are markers of improved health outcomes [16]. Liu et al. [17] showed that Roux-en-Y gastric bypass (RYGB) caused marked alterations in the gut microbiota and identified *Coriobacteriaceae* as a potential contributor to the beneficial effects on type 2 diabetes. The RYGB group was postoperatively enriched in Bacteroidetes, Fusobacteria and Actinobacteria compared to the sham surgery group and, based on the gut microbial patterns in the type 2 diabetes T2D patients, they found that *Coriobacteriaceae* within *Actinobacteria* might contribute to the beneficial effects of RYGB on T2D. In our study, the family *Coriobacteriaceae* and *B. thetaiotaomicron* may mediate the beneficial effects of kale under HFD conditions, particularly the attenuation of inflammation.

PICRUSt2 analysis revealed that the HFKV and LFD diets were similar in their effects on gut microbial metabolic functions. The HFKV diet attenuates the effects of the HFD on microbial function. HFKV highly enriched the glycan degradation and vitamin B1 metabolism activities of gut microbes. Glycans are attached to the backbone of proteins during structural modification through glycosylation. Glycosylation plays a role in cell to cell communication, intracellular signaling pathways and the modulation of the immune response [25] and regulates host immune responses, such as the reorganization of T cell receptor complexes, modulation of immune receptor clustering, endocytosis, receptor signaling and immunoglobulin functions [26]. Altered glycosylation patterns are reported in chronic inflammation and inflammatory disorders, including rheumatoid arthritis, inflammatory bowel disease, diabetes mellitus, cancer and infections [27]. Glycans without a terminal galactose induce the pro-inflammatory properties of immunoglobulin G and are observed in inflammatory diseases [28]. The observed anti-inflammatory effects of a kale diet under the HFD condition might be attributed to its increasing effects on gut microbial glycan degradation activities. The increased xenobiotic metabolism observed in HFKV-fed mice may enhance the metabolism of phytochemicals found in the vegetable, thus ensuring the metabolites are available to the host.

Thiamine is an essential factor for carbohydrate metabolism. Diabetic patients have thiamine deficiency, particularly in terms of plasma thiamine concentration. Pathophysiological causes of thiamine deficiency include oxidative stress, inflammation and endothelial dysfunction. High-dose thiamine treatment prevents diabetic-associated dyslipidemia in streptozotocin-induced diabetic rats [29] and thiamine and its active metabolites have anti-inflammatory properties through improved endothelial function and reduced oxidative stress in both the normo- and hyperglycemic conditions [30]. The significant impact of kale supplementation on gut microbial thiamine metabolism activity may be a contributing factor in its anti-inflammatory properties.

Metabolic function predictions demonstrated increased xenobiotic metabolism by cytochrome P450 (CYP450). This finding is in agreement with the study by Charron et al. [31], who reported that kale consumption increases the activity of CYP1A2, an isoform of CYP450. Because mammals recognize phytochemicals as xenobiotics, increased xenobiotic metabolism may ensure that the phytochemical metabolites of kale are available to the host. Xenobiotics, including a number of ligands produced directly and indirectly by the microbiome, are recognized by the aryl hydrocarbon receptor (AHR), an evolutionarily conserved receptor [31,32]. The metabolism of xenobiotic compounds is initiated by activation of the AHR, which translocates to the nucleus, where it acts as a transcription factor for target genes that include CYP1A1 and CYP1B [31]. The AHR is responsive to an array of xenobiotic compounds and induces the metabolism of exogenous compounds [31]. Broccoli has been shown to induce beneficial roles in intestinal health and inflammation through inducing the aryl hydrocarbon receptor (AHR) [32,33]. It is likely that the indole glucosinolate component in cruciferous vegetables mediates AHR activity. Besides xenobiotic metabolism, the physiological role of the AHR includes intestinal homeostasis [20].

Our study is limited because it did show causation and we did not determine the role of the individual phytochemical compounds present in kale. However, we show that whole kale has beneficial effects on the gut microbiome by increasing the bacterial diversity and proliferation of specific beneficial bacteria, such as *Coriobacteriaceae*. Furthermore, glycan degradation and vitamin B1 metabolism are enhanced by kale and these have been shown to play an important role in attenuating inflammation. A kale diet attenuates both HFD-induced reduction in gut microbe diversity and functional properties.

## Figures and Tables

**Table 1:** Nutrient analysis of leaves and stems of kale.

Component	Amount (%)	Analytical Methods Used
**Proximate Analysis**		
Ash	10.87	AOAC: 923.03
Carbohydrates	73.9	By calculation
Protein (6.25)	8.71	AACC 46–30; AOAC 992.15
Total Fat	1.88	AOAC: 996.06
**Fiber**		
Insoluble Dietary Fiber	37.8	AOAC: 991.43
Soluble Dietary Fiber	6.2	
Total Dietary Fiber	45.1	
**Fat**		AOAC: 996.06
Saturated Fat	0.28	
Monounsaturated Fat	0.21	
Cis-cis Polyunsaturated Fat	0.85	
Trans fat	0.46	
Moisture	4.65	AOAC: 945.43, 934.01
**Calories per 100 g**		
Calories	347.26	By calculation
Calories, 2020	184.74	
Calories from Fat	17	
Calories from Saturated Fat	3	
Calories (insoluble fiber subtracted)	196	

**Table 2:** Diet formulations used in this study.

Ingredients (g)	LFD	HFD	HFKV
Casein	200	200	191.88
L-Cystine	3	3	3
Corn starch	452.2	72.8	48.28
Maltodextrin 10	75	100	100
Sucrose	172.8	172.8	172.8
Cellulose	50	50	13.92
Soybean oil	25	25	23.46
Lard	20	177.5	177.5
Mineral mix	10	10	10
Dicalcium phosphate	13	13	13
Calcium carbonate	5.5	5.5	5.5
Potassium citrate	16.5	16.5	16.5
Vitamin mix	10	10	10
Choline bitartrate	2	2	2
Kale dried powdered leaves	0	0	82
**Total (g)**	**1055.05**	**858.15**	**869.89**
**Kcal from Specific Nutrients**			
Protein	716	716	716
Carbohydrate	2840	1422.4	1422.4
Fat	405	1822.5	1822.5
**Total Kcal**	**3961**	**3960.9**	**3961**

**Table 3:** (a) Obesity and inflammation markers after 12 weeks; (b) mRNA expression of inflammation markers in adipose tissue after 12 weeks.

**(a)**					
		**LFD**	**HFD**	**HFKV**	***p*-Value**
**Obesity Parameters**					
Calorie intake per day (Kcal/d)		11.2 (0.5) ^a^	11.5 (0.2) ^a^	11.4 (0.3) ^a^	ns
Body weight (g)		26.5 (1.8) ^a^	36.4 (2.2) ^b^	33.7 (3.8) ^b^	<0.05
Total body fat (g)		0.56 (0.12) ^a^	1.99 (0.23) ^b^	1.48 (0.2) ^b^	<0.05
HOMA-IR		0.11(0.03) ^a^	0.88 (0.18) ^b^	0.78 (0.15) ^b^	<0.05
**Serum Inflammation markers**					
MCP-1 (pg/mL)		776.1 (140.2) ^a^	1355.3 (140.2) ^b^	435.7 (140.2) ^c^	0.001
IL-10 (pg/mL)		195.3 (23.9) ^a^	284.7 (28.9) ^b^	332.9 (52.1) ^c^	<0.05
**(b)**					
**Marker**	**LFD**	**HFD**	**HFKV**	***p* Value**	**Primer Sequences**
TNFα	1.00 ^a^	0.81 (0.12) ^a^	0.21 (0.03) ^c^	<0.05	5′ TACTGAACTTCGGGGTGATTGGTCC 3′ 5′ CAGCCTTGTCCCTTGAAGAGAACC 3′
MCP-1	1.00 ^a^	14.9 (1.20) ^b^	3.23 (0.87) ^c^	<0.05	5′ CATCCACGTGTTGGCTCA 3′ 5′ AACTACAGCTTCTTTGGGACA 3′
CD11c	1.00 ^a^	2.51(0.30) ^b^	0.14 (0.02) ^c^	0.001	5′ CTGGATAGCCTTTCTTCTGCTG 3′ 5′ GCACACTGTGTCCGAACTC 3′
F4/80	1.00 ^a^	1.85 (0.03) ^b^	0.12(0.02) ^c^	0.001	5′ GGAAGGAAATGGAGAGAAAG 3′ 5′ GAAGATCTACCCTGGTGAAT 3′
IL-6	1.00 ^a^	2.84 (0.05) ^b^	0.27 (0.07) ^b^	0.001	5′ AGCCAGAGTCCTTCAGAGA 3′ 5′ TCCTTAGCCACTCCTTCTGT 3′
IL-10	1.00 ^a^	7.93 (0.04) ^b^	0.55 (0.04) ^c^	0.001	5′ GTCATCGATTTCTCCCCTGT 3′ 5′ ATGGCCTTGTAGACACCTT 3′
IL-8	nd	nd	nd	-	5′ ACAGAAAGGAAGTGATAGCAGT 3′ 5′ GCTTCATTGCCGGTGGAA3′
IL-1β	1.00 ^a^	0.12 (0.03) ^b^	0.16 (0.04) ^b^	<0.05	5′ CAACTGTTCCTGAACTCAACT 3′ 5′ ATCTTTTGGGGTCCGTCCAACT 3′
TLR4	1.00 ^a^	0.306 (0.02) ^b^	1.29 (0.02) ^c^	<0.05	5′ GACCGCAACAACGCCATCTA3′ 5′ GGCGTATCAGTGGGGGTCAG3′
NF-κB	1.00 ^a^	1.709 (0.02) ^b^	1.89 (0.03) ^b^	<0.05	5′ GCTGAGTCCTGCTCCTTCTAAA 3′ 5′ CCTCTGTGTAGCCCATCTGTTGC 3′
β-Actin					5′ CAGCTGAGAGGGAAATCGTG 3′ 5′ CGTTGCCAATAGTGATGACC 3′

Values represent mean and standard error (SE). Different letters in superscript indicate significant differences between the diet groups; ns = non-significant, nd = not detected.

**Table 4:** Different fermentation variables in three diet groups.

	LFD	HFD	HFKV	*p*-Value
Weight of full cecum (g)	0.35 (0.02)	0.311(0.03)	0.381 (0.03)	ns
Weight of empty cecum (g)	0.091 (0.03)	0.086 (0.002)	0.099 (0.003)	ns
pH of colon fecal samples	8.96 (0.14)	8.65 (0.02)	8.11 (0.15)	ns

Values under each diet group represent mean and standard error (SE); ns = non-significant.

**Table 5:** Relative abundance of different taxa between the 3 diets.

Taxa	LFD	HFD	HFKV	*p*-Value
**Firmicutes**				
**P-Firmicutes**	**57.3 ^a^**	**73.5 ^b^**	**63.8 ^c^**	**0.04**
**P-Firmicutes; C-Bacillus**	**17.9 ^a^**	**34.7 ^b^**	**19.4 ^a^**	**<0.005**
P-Firmicutes; C-Bacillus; O-Bacillales	3.6 ^a^	2.7 ^b^	2.4 ^b^	ns
**P-Firmicutes; C-Bacillus; O-Lactobacillales**	**14.3 ^a^**	**31.9 ^b^**	**17.0 ^a^**	**<0.005**
P-Firmicutes; C-Bacillus; O-Lactobacillales; F-Paenibacillaceae	3.5	3.2	2.7	ns
**P-Firmicutes; C-Bacillus; O-Lactobacillales; F-Lactobacilaceae**	**4.49 ^a^**	**14.3 ^b^**	**9.9 ^c^**	**<0.001**
P-Firmicutes; C-Bacillus; O-Lactobacillales; F-Lactobacilaceae; G-Acetatifactor	0.31	0.23	0.70	ns
**P-Firmicutes; C-Bacillus; O-Lactobacillales; F-Lactobacilaceae; G-Dorea**	**0.06**	**0.06**	**0.02**	**ns**
**P-Firmicutes; C-Bacillus; O-Lactobacillales; F-Lactobacilaceae; G-Blautia**	**0.11 ^a^**	**0.09 ^a^**	**0.28 ^b^**	**<0.05**
**P-Firmicutes; C-Bacillus; O-Lactobacillales; F-Lactobacilaceae; G-Roseburia**	**0.04 ^a^**	**0.05 ^a^**	**0.48 ^b^**	**<0.05**
P-Firmicutes; C-Bacillus; O-Lactobacillales; F-Lactobacilaceae; G-Tyzerella	0.03 ^a^	0.05	0.0 ^a^	ns
**P-Firmicutes; C-Bacillus; O-Lactobacillales; F-Lactobacilaceae; G-Lactobacillus**	**0.48 ^a^**	**7.6 ^b^**	**3.3 ^c^**	**0.001**
**P-Firmicutes; C-Bacillus; O-Lactobacillales; F-Lactobacilaceae; G-Lactobacillus; S-*L. johnsonii***	**0.24 ^a^**	**3.06 ^b^**	**1.04 ^c^**	**<0.05**
**P-Firmicutes; C-Bacillus; O-Lactobacillales; F-Streptococaceae**	**13.8 ^a^**	**24.0 ^b^**	**14.6 ^a^**	**0.04**
**P-Firmicutes; C-Bacillus; O-Lactobacillales; F-Streptococaceae; G-Lactococcus**	**6.72 ^a^**	**14.4 ^b^**	**7.85 ^a^**	**0.04**
**P-Firmicutes; C-Bacillus; O-Lactobacillales; F-Streptococaceae; G-Lactococcus; S-*L. lactis***	**6.19 ^a^**	**8.41 ^b^**	**5.91 ^a^**	**<0.05**
**P-Firmicutes; C-Erysipelotrichia**	**18.8 ^a^**	**18.3 ^a^**	**15.1 ^b^**	**<0.05**
**P-Firmicutes; C-Erysipelotrichia; O-Erysipelotrichales**	**18.7 ^a^**	**17.5 ^a^**	**14.9 ^b^**	**ns**
P-Firmicutes; C-Erysipelotrichia; O-Erysipelotrichales; F-Erysipelotrichaceae	18.7 ^a^	10.3 ^b^	13.1 ^b^	ns
P-Firmicutes; C-Erysipelotrichia; O-Erysipelotrichales; F-Erysipelotrichaceae; G-*Clostridium*	0.07	0.13	0.08	ns
P-Firmicutes; C-Erysipelotrichia; O-Erysipelotrichales; F-Erysipelotrichaceae; G-*Turicibacter*	1.98	2.71	2.55	ns
P-Firmicutes; C-Clostridia	20.7	21.4	24.1	ns
P-Firmicutes; C-Clostridia; O-Clostridiales	20.7	21.4	24.3	ns
P-Firmicutes; C-Clostridia; O-Clostridiales; F-Eubacteriaceae	0.20	0.22	0.26	ns
**P-Firmicutes; C-Clostridia; O-Clostridiales; F-Eubacteriaceae; G-Eubactrium**	**0.01 ^a^**	**0.02 ^a^**	**0.39 ^b^**	**0.04**
**P-Firmicutes; C-Clostridia; O-Clostridiales; F-Lachnospiraceae**	**9.5 ^a^**	**11.3 ^b^**	**13.2 ^b^**	**0.04**
**P-Firmicutes; C-Clostridia; O-Clostridiales; F-Peptostreptococcaceae**	**0.56 ^a^**	**1.67 ^b^**	**1.75 ^b^**	**0.04**
P-Firmicutes; C-Clostridia; O-Clostridiales; F-Oscillospiraceae	0.35	0.22	0.10	ns
P-Firmicutes; C-Clostridia; O-Clostridiales; F-Ruminococaceae	3.74	4.70	5.64	ns
P-Firmicutes; C-Clostridia; O-Clostridiales; F-Ruminococaceae; G-Ruminococcus	3.43	4.33	5.06	ns
P-Firmicutes; C-Clostridia; O-Clostridiales; F-Ruminococaceae; G-Ruminococcus; S-*R. gnavus*	2.60	3.29	3.55	ns
P-Firmicutes; C-Clostridia; O-Clostridiales; F-Ruminococaceae; G-Faecalibacterium	0.31	0.37	0.64	ns
P-Firmicutes; C-Clostridia; O-Clostridiales; F-Ruminococaceae; G-Faecalibacterium; S-*F. prausnitzii*	0.07	0.09	0.03	ns
P-Firmicutes; C-Clostridia; O-Clostridiales; F-Clostridiaceae	7.66	6.17	8.44	ns
P-Firmicutes; C-Clostridia; O-Clostridiales; F-Clostridiaceae; G-Clostridium	7.51	5.94	7.81	ns
P-Firmicutes; C-Clostridia; O-Clostridiales; F-Clostridiaceae; G-Clostridium; *S-C. vincentii*	3.01	2.08	2.38	ns
P-Firmicutes; C-Clostridia; O-Clostridiales; F-Clostridiaceae; G-Clostridium; S-*C. disporicum*	0.52	0.52	0.35	ns
P-Firmicutes; C-Clostridia; O-Clostridiales; F-Clostridiaceae; G-Clostridium; S-*C. scindens*	0.17	0.15	0.08	ns
P-Firmicutes; C-Clostridia; O-Clostridiales; F-Clostridiaceae; G-Clostridium; S-*C. hiranonis*	0.10	0.34	0.42	ns
**Bacteroidetes**				
**P-Bacteroidetes**	**24.2 ^a^**	**14.4 ^b^**	**24.5 ^a^**	**0.01**
**P-Bacteroidetes; C-Cytophagia**	**1.02 ^a^**	**0.31 ^b^**	**0.35 ^b^**	**<0.05**
P-Bacteroidetes; C-Sphingobacteria	0.03	0.03	0.04	ns
**P-Bacteroidetes; C-Flavobacteria**	**1.62 ^a^**	**2.93 ^b^**	**0.93 ^c^**	**<0.05**
P-Bacteroidetes; C-Flavobacteria; O-Flavobacteriales	1.16	0.83	0.87	ns
**P-Bacteroidetes; C-Flavobacteria; O-Flavobacteriales; F-Flavobacteraceae**	**1.6 ^a^**	**2.32 ^b^**	**0.52 ^c^**	**<0.05**
**P-Bacteroidetes; C-Bacteroidia**	**20.7 ^a^**	**18.7 ^b^**	**23.1 ^a^**	**0.01**
**P-Bacteroidetes; C-Bacteroidia; O-Bacteroidales**	**20.7 ^a^**	**17.6 ^b^**	**23.1 ^a^**	**<0.05**
**P-Bacteroidetes; C-Bacteroidia; O-Bacteroidales; F-Bacteroidaceae**	**13.9 ^a^**	**12.91 ^a^**	**15.9 ^b^**	**<0.05**
**P-Bacteroidetes; C-Bacteroidia; O-Bacteroidales; F-Bacteroidaceae; G-Bacteroides**	**7.53 ^a^**	**6.88 ^b^**	**9.76 ^b^**	**0.01**
**P-Bacteroidetes; C-Bacteroidia; O-Bacteroidales; F-Bacteroidaceae; G-Bacteroides; S-*B. thetamicron***	**6.48 ^a^**	**5.69 ^b^**	**8.86 ^c^**	**<0.01**
P-Bacteroidetes; C-Bacteroidia; O-Bacteroidales; F-Porphyromonadaceae	3.84	4.16	3.32	ns
**P-Bacteroidetes; C-Bacteroidia; O-Bacteroidales; F-Prevotallaceae**	**2.39** ^a^	**1.57** ^b^	**2.91** ^a^	**<0.05**
P-Bacteroidetes; C-Bacteroidia; O-Bacteroidales; F-Rikenellaceae	0.64	1.24	0.97	ns
**P-Bacteroidetes; C-Bacteroidia; O-Bacteroidales; F-Rikenellaceae; G-Alistipes**	**0.44 ^a^**	**0.51 ^a^**	**0.83 ^b^**	**0.01**
**Actinobacteria**				
**P-Actinobacteria**	**21.2 ^a^**	**8.9 ^b^**	**11.2 ^b^**	**0.001**
**P-Actinobacteria; C-Actinobacteria**	**20.0 ^a^**	**8.6 ^b^**	**10.8 ^b^**	**0.001**
P-Actinobacteria; C-Actinobacteria; O-Actinomycetales	0.07	0.03	0.05	ns
**P-Actinobacteria; C-Actinobacteria; O-Bifidobacteriales**	**18.5 ^a^**	**7.72 ^b^**	**7.43 ^b^**	**0.001**
**P-Actinobacteria; C-Actinobacteria; O-Bifidobacteriales; F-Bifidobacteroidaceae**	**18.6 ^a^**	**7.72 ^b^**	**7.43 ^b^**	**0.001**
**P-Actinobacteria; C-Actinobacteria; O-Bifidobacteriales; F-Bifidobacteroidaceae; G-Bifidobacterium**	**17.8 ^a^**	**7.34 ^b^**	**7.09 ^b^**	**0.001**
**P-Actinobacteria; C-Actinobacteria; O-Bifidobacteriales; F-Bifidobacteroidaceae; G-Bifidobacterium; S-*B. pseudolongum***	**12.6 ^a^**	**5.49 ^b^**	**5.73 ^b^**	**0.001**
**P-Actinobacteria; C-Actinobacteria; O-Bifidobacteriales; F-Bifidobacteroidaceae; G-Bifidobacterium; S-*B. choerinum***	**0.41 ^a^**	**0.15 ^b^**	**0.10 ^b^**	**0.001**
**P-Actinobacteria; C-Coriobacteriia**	**1.34 ^a^**	**0.89 ^a^**	**3.40 ^c^**	**<0.001**
**P-Actinobacteria; C-Coriobacteriia; O-Coriobacteriales**	**1.39 ^a^**	**0.87 ^a^**	**3.38 ^c^**	**<0.001**
**P-Actinobacteria; C-Coriobacteriia; O-Coriobacteriales; F-*Coriobacteriaceae***	**1.39 ^a^**	**0.87 ^a^**	**3.38 ^c^**	**<0.001**
**P-Actinobacteria; C-Coriobacteriia; O-Coriobacteriales; F-*Coriobacteriaceae*; G-Atopobium**	**0.67 ^a^**	**0.26 ^a^**	**1.26 ^c^**	**<0.05**
**P-Actinobacteria; C-Coriobacteriia; O-Coriobacteriales; F-*Coriobacteriaceae*; G-Enterorhabdus**	**0.04 ^a^**	**0.03 ^a^**	**0.25 ^b^**	**0.05**
**P-Actinobacteria; C-Coriobacteriia; O-Coriobacteriales; F-*Coriobacteriaceae*; G-Enterorhabdus; S-*E. caecimuris***	**0.04 ^a^**	**0.02 ^a^**	**0.19 ^b^**	**0.05**
**Proteobacteria**				
P-Proteobacteria	0.68	0.22	0.31	ns
P-Proteobacteria; C-Betaproteobacteria	0.58	0.16	0.17	ns
P-Proteobacteria; C-Betaproteobacteria; O-Bukholderiales	0.56	0.16	0.13	ns
P-Proteobacteria; C-Betaproteobacteria; O-Bukholderiales; F-Sutterellaceae	0.56	0.13	0.16	ns
P-Proteobacteria; C-Betaproteobacteria; O-Bukholderiales; F-Sutterellaceae; G-Parasutterella	0.55	0.12	0.14	ns
P-Proteobacteria; C-Betaproteobacteria; O-Bukholderiales; F-Sutterellaceae; G-Parasutterella; S-*P. excrementihominis*	0.45	0.11	0.12	ns
P-Proteobacteria; C-Betaproteobacteria; O-Neisseriales	0.05	0.1	0.1	ns
P-Proteobacteria; C-Betaproteobacteria; O-Neisseriales; F-Neisseriaceae	0.05	0.1	0.1	ns
**P-Proteobactetria; C-Gammaproteobacteria**	**0.02 ^a^**	**1.63 ^b^**	**0.73 ^c^**	**0.04**
**P-Proteobactetria; C-Gammaproteobacteria; O-Enterobacterales**	**0.02 ^a^**	**1.63 ^b^**	**0.69 ^b^**	**0.04**
**P-Proteobactetria; C-Gammaproteobacteria; O-Enterobacterales; F-Enterobacteriaceae**	**0.02 ^a^**	**1.63 ^b^**	**0.69 ^b^**	**0.04**
**Verrucomicrobia**				
**P-Verrucomicrobia**	**0.06 ^a^**	**0.24 ^b^**	**0.22 ^b^**	**<0.05**
**P-Verrucomicrobia; G-Akkermansia**	**0.04 ^a^**	**0.22 ^b^**	**0.21 ^b^**	**<0.05**
**P-Verrucomicrobia; G-Akkermansia; S-*A. municiphila***	**0.04 ^a^**	**0.22 ^b^**	**0.21 ^b^**	**<0.05**

Values represent means. Different letters in superscript indicate significant differences between groups. ns = non-significant. All values in bold are significant.

## Data Availability

All data generated or analyzed during this study are included in this published article. There are no Supplementary Information files.
